# Anatomic and Physiologic Heterogeneity of Subgroup-A Auditory Sensory Neurons in Fruit Flies

**DOI:** 10.3389/fncir.2017.00046

**Published:** 2017-06-28

**Authors:** Yuki Ishikawa, Natsuki Okamoto, Mizuki Nakamura, Hyunsoo Kim, Azusa Kamikouchi

**Affiliations:** Division of Biological Science, Graduate School of Science, Nagoya UniversityNagoya, Japan

**Keywords:** Johnston's organ, *Drosophila*, Ca^2+^ imaging, mechanosensory, insect, auditory behavior, courtship song, acoustic communication

## Abstract

The antennal ear of the fruit fly detects acoustic signals in intraspecific communication, such as the courtship song and agonistic sounds. Among the five subgroups of mechanosensory neurons in the fly ear, subgroup-A neurons respond maximally to vibrations over a wide frequency range between 100 and 1,200 Hz. The functional organization of the neural circuit comprised of subgroup-A neurons, however, remains largely unknown. In the present study, we used 11 *GAL4* strains that selectively label subgroup-A neurons and explored the diversity of subgroup-A neurons by combining single-cell anatomic analysis and Ca^2+^ imaging. Our findings indicate that the subgroup-A neurons that project into various combinations of subareas in the brain are more anatomically diverse than previously described. Subgroup-A neurons were also physiologically diverse, and some types were tuned to a narrow frequency range, suggesting that the response of subgroup-A neurons to sounds of a wide frequency range is due to the existence of several types of subgroup-A neurons. Further, we found that an auditory behavioral response to the courtship song of flies was attenuated when most subgroup-A neurons were silenced. Together, these findings characterize the heterogeneous functional organization of subgroup-A neurons, which might facilitate species-specific acoustic signal detection.

## Introduction

Acoustic information is important for animal survival and reproduction in many species. To recognize the temporal pattern of informative acoustic sounds, many animals have evolved a dedicated receptor organ and its downstream neural circuits. The anatomy of the downstream neural circuits are systematically organized so that the central nervous system represents the stimulus features, such as the frequency, direction, and temporal pattern, as a topographic map (Hildebrandt, 2014).

Fruit flies, *Drosophila melanogaster* and its related species, utilize acoustic signals for intraspecific communication. During courtship, male flies vibrate their wings, producing a courtship song comprising a continuous sine song and an intermittent pulse song. The temporal patterns of courtship songs, especially the interpulse interval (IPI) in the pulse song, vary among related species (Ewing and Bennet-Clark, 1968; Cowling and Burnet, 1981). Species-specific pulse songs effectively accelerate the females' receptivity for copulation and the courtship behavior of males in *D. melanogaster* (Ritchie et al., 1999; Yoon et al., 2013; Zhou et al., 2015), suggesting that the auditory system of fruit flies can distinguish the conspecific IPI.

Fruit flies detect sounds with a pair of antennal mechanosensory organs, Johnston's organ (JO), located within the second segment of the antenna. Five subgroups of sensory neurons, JO neurons, and support cells make up the JO (Kamikouchi et al., 2006). Subgroup A, B, and D-JO neurons (JO-A, JO-B, and JO-D neurons, respectively) are strongly activated by antennal vibrations and are thus referred to as the auditory sensory neurons in fruit flies (Kamikouchi et al., 2009; Yorozu et al., 2009). These three subgroups have distinct response characteristics; JO-A neurons preferentially respond to high frequency vibrations (>100 Hz), while JO-B neurons selectively respond to low frequency sound (<100Hz) and JO-D neurons are highly activated by vibrations of a middle-range frequency (100–200 Hz; Kamikouchi et al., 2009; Yorozu et al., 2009; Matsuo et al., 2014). Previous studies identified a central auditory pathway that controls courtship behavior, which starts in JO-B neurons and proceeds to the AMMC-B1 (aPN1), vPN1, and pC1 neurons (Kamikouchi et al., 2009; Zhou et al., 2015). The function of JO-A neurons, however, is unknown, except for their role in the mechanical amplification of antennal vibrations to faint sound (Kamikouchi et al., 2009; Effertz et al., 2011).

**Figure 1:**
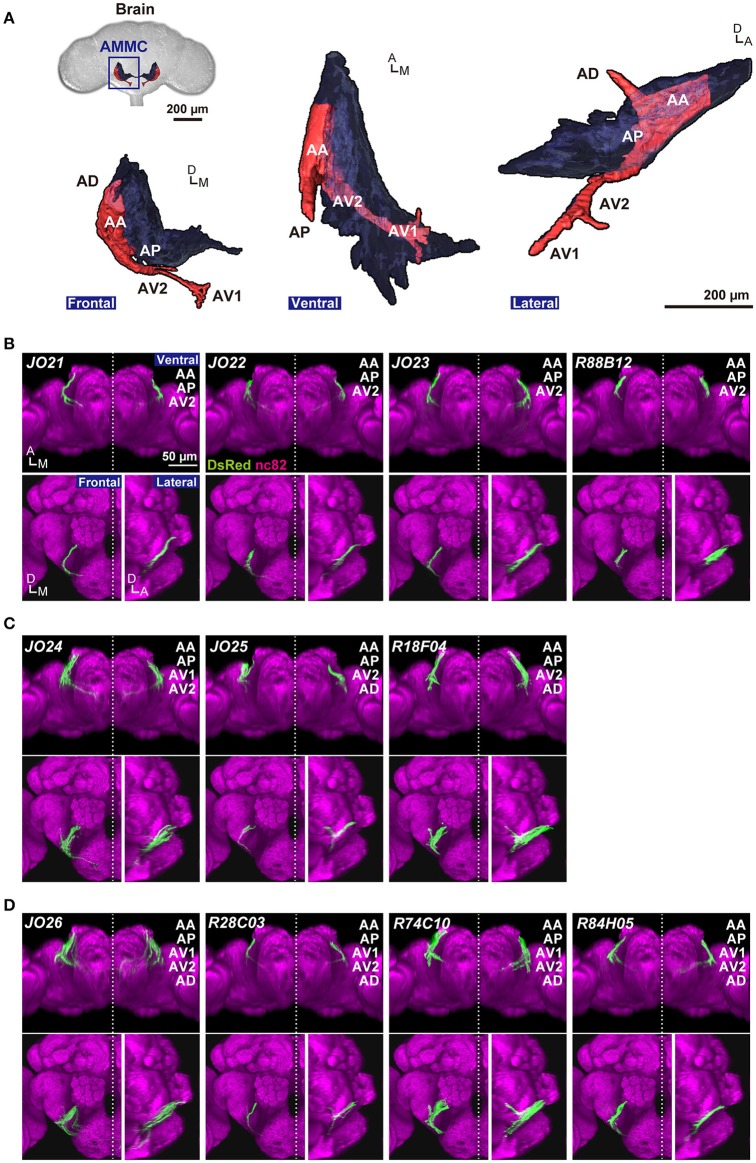
Anatomy of JO-A neurons. **(A)** Frontal, ventral, and lateral views of 3D-reconstructed AMMC zone A in a fly brain. Zone A and other AMMC zones are shown in red and black, respectively. Five subareas in the zone A (subareas AA, AP, AV1, AV2, and AD) represent the projection target of JO-A neurons in the brain. **(B–D)** *GAL4* strains that label JO-A neurons (*JO-A GAL4* strains). Labeled subareas in each strain are shown at the upper-right of the panel. Ventral, frontal, and lateral views of the brain of each strain that labels three **(B)**, four **(C)**, and five subareas **(D)** are shown. All images were registered to a template brain. Green and magenta signals show the DsRed-labeled neurons and nc82-labeled neuropils, respectively. Signals of cells that were not relevant to JO-A neurons were manually erased from the original images for clarity. Original labeling patterns are shown for *JO* strains (Kamikouchi et al., 2006) and FlyLight database (http://flweb.janelia.org/cgi-bin/flew.cgi) for *FlyLight* strains. A, anterior; D, dorsal; M, medial. Images in panel **(A)** were modified from Ishikawa and Kamikouchi (2016) with permission.

The projection target of JO-A neurons is located in the lateral part of the antennal mechanosensory and motor center (AMMC) in the brain, called AMMC zone A. AMMC zone A is anatomically divided into five subareas, AA, AP, AD, AV1, and AV2, in which the distal tip of subareas AV1 and AD overlap with the gnathal ganglia and wedge, respectively (Kamikouchi et al., 2006; Figure 1A). A previous study demonstrated that the anatomy of single JO-A neurons is quite diverse; at least 13 “types” of JO-A neurons have been identified, each of which is defined by its distinct projection pattern to the five subareas in zone A (Kamikouchi et al., 2006). Whether these anatomically diverse neurons also have heterogeneous response properties and functions for auditory behavior, however, remains unknown.

Here, we explored the heterogeneity of JO-A neurons at the anatomic, physiologic, and behavioral levels to understand the organization of auditory pathway contributed by JO-A neurons. By using 11 *GAL4* strains that selectively label subgroup-A neurons, we found that the anatomic heterogeneity of JO-A neurons was more diverse than previously reported, and the axonal projection patterns in the brain of some JO-A neurons correlated with the somata location in the JO. We also evaluated the physiologic heterogeneity of these neurons by observing the increase in Ca^2+^ in particular subsets of JO-A neurons in response to antennal vibrations of various frequencies and IPIs. Finally, we demonstrate that the functions of all JO-A neurons together might be important in sound-induced chaining behavior.

## Materials and methods

### Experimental animals

Fruit flies *D. melanogaster* were raised on standard yeast-based media at 25°C and 40–60% relative humidity. The following transgenic *GAL4*-driver strains were used for the GAL4/UAS (Brand and Perrimon, 1993) and FLP-out (Basler and Struhl, 1994) techniques: *F-GAL4* (Kim et al., 2003), *iav-GAL4* (Kwon et al., 2010), *JO15* (Sharma et al., 2002), *JO21, JO22, JO23, JO24, JO25, JO26* (*NP0799, NP1316, NP3595, NP1017, NP5021, NP1109*, respectively, in KYOTO Stock Center, Kyoto, Japan; Kamikouchi et al., 2006), *R18F04, R28C03, R74C10, R84H05*, and *R88B12* (the FlyLight collection; RRID: BDSC_48820, 49448, 39848, 40405, and 46851, respectively; Jenett et al., 2012), and *vGluT-GAL4* (Bloomington Stock Center; RRID: BDSC_24635). The following *UAS-reporter* strains were used: *UAS-DsRed S197Y* (Verkhusha et al., 2001) to label GAL4-positive neurons, *UAS-GCaMP3* (for *F-GAL4*) and *UAS-GCaMP6m* (for other *JO-A GAL4* strains; Bloomington Stock Center, Bloomington, IN; RRID: BDSC_32236 and 42748) for the Ca^2+^ imaging and *UAS*-*RedStinger* (Bloomington Stock Center, Bloomington, IN; RRID: BDSC_8546 and 8547; Barolo et al., 2004) to visualize labeled cell nucleus of JO neurons. Flies carrying the transgenes *hs*-*flp, UAS*>*CD*2, *y*^+^ >*CD8::GFP* (RRID: BDSC_56799; Wong et al., 2002) and *UAS-IVS-mCD8::RFP* (Bloomington Stock Center; RRID: BDSC_32219) were used for the FLP-out analysis. To induce flippase expression, adult flies from 1 to 3 days after eclosion or third instar larvae in a plastic tube were placed for 5–30 min in a water bath at 37°C (adult flies) or 39°C (larvae). Optimal heat-shock condition to induce single-cell expression varied between strains (Table 1). Female flies 5–10-day old after eclosion were used in the immunohistochemical analysis. For Ca^2+^ imaging, 2–12-day old female flies were used.

**Table 1:** Optimal heat-shock condition in each *GAL4* strain.

**Strain**	**Condition**	**Adult (37°C, min)**							
		**0**	**10**	**15**	**30**	**45**	**60**	**75**	**90**
*JO21 (NP0799)*	FLP-out	0	0	1	1	4	4	2	2
	Single	0	0	1	0	1	0	0	0
	Total	11	14	15	12	15	12	9	6
*JO22 (NP1346)*	FLP-out	0	0	5	4	4	4	n/a	n/a
	Single	0	0	4	0	2	0	n/a	n/a
	Total	7	11	8	12	10	8	n/a	n/a
*JO23 (NP3595)*	FLP-out	1	0	2	4	9	7	n/a	n/a
	Single	0	0	2	3	0	0	n/a	n/a
	Total	8	9	13	10	12	12	n/a	n/a
*JO24 (NP1017)*	FLP-out	n/a	0	4	8	n/a	n/a	n/a	n/a
	Single	n/a	0	3	5	n/a	n/a	n/a	n/a
	Total	n/a	8	12	9	n/a	n/a	n/a	n/a
*JO25 (NP5021)*	FLP-out	1	0	3	6	7	n/a	n/a	n/a
	Single	0	0	1	2	0	n/a	n/a	n/a
	Total	4	3	17	9	12	n/a	n/a	n/a
*JO26 (NP1109)*	FLP-out	n/a	0	0	8	n/a	n/a	n/a	n/a
	Single	n/a	0	0	4	n/a	n/a	n/a	n/a
	Total	n/a	8	5	8	n/a	n/a	n/a	n/a
*JO15*	FLP-out	1	n/a	6	8	n/a	n/a	n/a	n/a
	Single	1	n/a	0	0	n/a	n/a	n/a	n/a
	Total	8	n/a	7	8	n/a	n/a	n/a	n/a
**Strain**	**Condition**	**Larva (39°C, min)**			**Adult (37°C, min)**				
		**15**		**30**	**0**		**15**	**30**	
*R18F04*	FLP-out	3		0	2		3	7	
	Single	0		0	2		3	1	
	Total	4		1	8		9	8	
*R28C03*	FLP-out	15		4	n/a		0	0	
	Single	3		2	n/a		0	0	
	Total	18		11	n/a		9	8	
*R74C10*	FLP-out	9		8	1		9	8	
	Single	1		1	1		4	0	
	Total	9		9	10		10	10	
*R84H05*	FLP-out	n/a		0	0		6	5	
	Single	n/a		0	0		4	2	
	Total	n/a		9	10		13	7	
*R88B12*	FLP-out	4		9	n/a		2	9	
	Single	0		2	n/a		1	1	
	Total	5		8	n/a		10	10	

*NP-series and JO15 GAL4 strains are heat-shocked at 37°C at their adult stage. FlyLight strains are heat-shocked at 39°C at their 3rd-instar larva or 37°C at the adult stage. n/a, not analyzed*.

For behavioral assays, flies carrying the transgenes *UAS-TNT* and *UAS-IMPTNT* (RRID: BDSC_28838 and BDSC_28840; Sweeney et al., 1995) were used as effector strains and their controls, respectively. After crossing them with a *GAL4* strain, adult virgin males were collected within 8 h of eclosion; their wings were clipped on the same day following brief induction of anesthesia on ice. Flies between 5 and 7 days after eclosion were used.

### Immunohistochemistry

Immunolabeling of the antennae and brains was performed as described previously (Matsuo et al., 2016). When labeled with anti-glutamate antibody, tissues were fixed in Zanboni's fixative for 60 min on ice. For immunolabeling with other antibodies, antennae and brains were fixed with 4% formaldehyde in PBS for 2 h at 4°C or 4% paraformaldehyde in PBS for 90 min on ice, respectively. Primary antibodies used in this study were as follows: Rat anti-Elav (DSHB, Iowa City, IA, #RAT-Elav-7E8A10, RRID: AB_528218, used at 1:250 dilution) to label nuclei of neurons. Rabbit anti-DsRed (Living Colors DsRed Polyclonal Antibody; Clontech, Mountain View, CA, #632496, RRID: AB_10013483, used at 1:1,000 dilution) for enhancing the signals of DsRed, mCD8::RFP, and RedStinger. Rabbit anti-GFP (Invitrogen, La Jolla, CA, #A11122, RRID: AB_221569, used at 1:1,000 dilution) and rat anti-GFP (Nacalai Tesque, Japan, #04404-26, used at 1:1,000 dilution) for enhancing the CD8::GFP signals. Mouse anti-Bruchpilot nc82 (DSHB, #nc82, RRID: AB_528108, used at 1:20 dilution; donated by Buchner, E.) to visualize synaptic regions in the brain. Rabbit anti-serotonin (5-hydroxytryptamine, 5-HT; Immunostar, #20080, used at 1:300 dilution), mouse anti-glutamate (Sigma Aldrich, #G9282, used at 1:20 dilution), mouse anti-Choline acetyltransferase (ChAT; DSHB, #ChAT4B1-s, used at 1:10 dilution; donated by Salvaterra, P. M.), and rabbit anti-gamma amino butyric acid (GABA; Sigma Aldrich, #A2052, used at 1:300 dilution) to verify neurotransmitters expressed in JO neurons. Rabbit anti-Tetanus-Toxin (Statens Serum Institut, Copenhagen S, Denmark, POL 016, used at 1:1,000 dilution) to detect Tetanus toxin (TNT) expressed in JO neurons.

Secondary antibodies used in this study were as follows: Alexa Fluor 488-conjugated anti-rat IgG (Jackson ImmunoResearch, Dianova, Göttingen, Germany, #112-545-167; used at 1:300 dilution), Alexa Fluor 488-conjugated anti-rabbit IgG (Invitrogen, A11034; used at 1:300 dilution), Alexa Fluor 555-conjugated anti-rat IgG (Invitrogen, A21434; used at 1:300 dilution), Alexa Fluor 555-conjugated anti-rabbit IgG (Invitrogen, #A21429, RRID: AB_141761, used at 1:300 dilution), Alexa Fluor 647-conjugated anti-mouse IgG (Invitrogen, #A21236, RRID: AB_141725, used at 1:300 dilution), and Alexa Fluor 647-conjugated anti-rat IgG (Jackson ImmunoResearch, #112-605-167, used at 1:300 dilution).

### Confocal microscopy and image processing

Serial optical sections of the antennae and brains were obtained at 0.84-μm (brains) or 0.57-μm (antennae) intervals with an FV-1000D laser-scanning confocal microscope (Olympus, Tokyo, Japan) equipped with a silicone-oil immersion 30x (brains) or 60x (antennae) Plan-Apochromat objective lens (NA = 1.05 and 1.3, respectively). For three-dimensional (3D) image reconstruction, confocal image datasets were processed with the 3D-reconstruction software FluoRender (http://www.fluorender.org; Wan et al., 2009). For the projection analysis and FLP-out image analysis, signals of cells that were not relevant to the traced neurons were erased manually from the original images with FluoRender for clarity (Figures 1, **3**). For the images of axonal projections of labeled JO neurons in the brain, cleaned image stacks were aligned to a template brain with non-rigid registration using the Computational Morphometry Toolkit (CMTK; Jefferis et al., 2007). To visualize the somata locations of single JO-A neurons that innervate specific subarea(s), the location of each somata was mapped manually onto the somata array of JO neurons according to the corresponding confocal image (**Figure 5**). The size, contrast, and brightness of the images were adjusted using Photoshop CS5 or later (Adobe Systems, San Jose, CA).

### Ca^2+^ imaging

Ca^2+^ imaging was performed as described previously with minor modifications (Matsuo et al., 2014). Briefly, flies were anesthetized on ice and affixed onto an imaging plate using silicon grease (SH 44M, Toray, Tokyo, Japan) with the ventral side of the fly up. The mouthpart of the fly was then removed using fine tweezers to open a window through which we could monitor brain fluorescence. A drop of phosphate buffered saline (PBS) or hemolymph-like saline solution (Lai et al., 2012) was immediately added to prevent dehydration. A fluorescent microscope (Axio Imager.A2; Carl Zeiss, Oberkochen, Germany) equipped with a water-immersion 20x objective lens (N.A. = 0.5), a spinning disc confocal head CSU-W1 (Yokogawa, Tokyo, Japan), and an OBIS 488 LS laser (Coherent, Santa Clara, CA) for excitation at 488 nm was used. The fluorescent image was captured at a rate of 4 Hz (for frequency analysis) or 10 Hz (for pulse-song analysis) with an exposure time of 250 or 100 ms using an EM-CCD camera (ImagEM512, Hamamatsu Photonics, Shizuoka, Japan) in water-cooled mode. Each experiment was performed in at least five flies.

### Imaging data analysis

Image data were analyzed offline with ImageJ (National Institutes of Health), Excel (Microsoft), and R software (https://www.r-project.org). Images were corrected for the animal's movement using the ImageJ plug-in TurboReg (http://bigwww.epfl.ch/thevenaz/turboreg/). We selected regions of interest for each subarea of zone A where abundant output synapses were observed (Kamikouchi et al., 2006). The GCaMP3 and GCaMP6m fluorescence intensities (ΔF/F_0_) were normalized to those preceding the stimulus onset (*t* = −2 s). In the pulse-song analysis, ΔF/F_0_ was adjusted by fitting the exponential decay function [y = a^*^exp(−bx) + c] to exclude the bleaching effect; the constants were calculated using ImageJ from the ΔF/F_0_ data obtained during the 10 frames before the stimulus and 18 frames after the stimulus (from 2.3 to 4.0 s after the stimulus period). To compare the response properties between *GAL4* strains, ΔF/F_0_ intensity was normalized with the maximum ΔF/F_0_ intensity in each individual (normalized ΔF/F_0_). As the pulse number in a single frame varied from frame to frame and among sound stimuli, normalized ΔF/F_0_ was calculated by dividing ΔF/F_0_ by the pulse number in each frame.

For comparison of the response property among *GAL4* strains, Scheirer-Ray-Hare test, a non-parametric alternative to two-factor ANOVA with replication, was performed. For comparison of normalized ΔF/F_0_ among sound stimuli in each *GAL4* strain, Friedman test followed by *post-hoc* Wilcoxon-Nemenyi-McDonald-Thompson test was performed. All statistical analyses were performed by R software. Friedman test with Wilcoxon-Nemenyi-McDonalds-Thompson test were applied, using the R code of “Tal Galili” (from https://www.r-statistics.com/2010/02/post-hoc-analysis-for-friedmans-test-r-code).

### Electrostatic actuation of the antennal receiver

Antennal displacement was induced by electrostatic force (Albert et al., 2007; Kamikouchi et al., 2009). The electrical potential of the fly was increased to +14 V against ground via a charging electrode (tungsten wire of 0.03 mm in diameter or platinum wire of 0.3 mm in diameter, The Nirako Corporation, Tokyo, Japan) inserted into the thorax. Voltage commands ranging from −14 V to +14 V were fed for 4 s in sinusoids (40, 100, 200, 400, 800 Hz) and 20 pulses in pulse-song like vibrations (intra-pulse frequency = 167 Hz; IPI = 15, 35, 55, 75, 95, 105 ms) to a stimulus probe (platinum wire of 0.3 mm in diameter, The Nirako Corporation, Tokyo, Japan) placed in front of the arista, the antennal receiver of the fruit fly. These electrical signals were generated with a data acquisition unit (Micro1401, Cambridge Electronic Design, Cambridge, UK) operated by Spike2 software (Cambridge Electronic Design), amplified by a custom-made amplifier, and fed into a stimulus probe. The peak-to-peak amplitude of the stimulus-induced vibration, measured by using a Polytec NLV-2500 scanning laser Doppler vibrometer with a VIB-A-20xLENS close-up lens (Polytec Japan, Yokohama, Japan; Matsuo et al., 2014), was ~1.31 ± 0.52 μm (mean ± standard deviation) throughout the experiment.

### Behavioral assay

Behavioral response of flies to sound was performed as described (Yoon et al., 2013). Briefly, six males were transferred gently to each behavioral chamber and placed in front of a loudspeaker (TAMON, Japan). Artificial pulse song used as an acoustic stimulus comprises the repetition of 1-s pulse burst and a subsequent 2-s pause, in which the pulses in the pulse burst have 35-ms IPI and 167-Hz intra-pulse frequency. The duration of a single pulse in the pulse burst is about 6 ms. Mean baseline-to-peak amplitude of its particle velocity is 9.2 mm/s. The sound starts 5 min after the video-recording onset and lasts for 6.5 min as described (Yoon et al., 2013). Video recording was performed using a monochrome digital camera (Himawari GE60, Library, Tokyo, Japan) equipped with a zoom lens (Lametar 2.8/25 mm, Jenoptik GmbH, Jena, Germany).

Chaining behavior was analyzed with ChaIN software (Yoon et al., 2013) with minor modifications. Previously, we counted the number of all the flies in male-male courtship chains (Eberl et al., 1997; Yoon et al., 2013; Zhou et al., 2015). In the present study, we counted only the followers in each chain, as the fly at the front of each chain is a passive recipient of the male-male courtship chain.

Statistical analysis was performed using R software. Because the Shapiro-Wilk normality tests revealed significant differences in several categories (e.g., *p* = 0.0011 in increase of chain index in *R74C10* flies), the Wilcoxon signed-rank test and Wilcoxon rank sum test were applied to compare chain indices between before and after sound stimulus, and the increase of chain indices between groups, respectively (**Table 7**). Because the analyses included multiple comparisons, alpha levels were corrected by a modified “step-down” procedure of Benjamini and Hochberg method to keep the false discovery rate below 5% (Benjamini and Yekutieli, 2001; Guo and Rao, 2008).

## Results

### Screening of *GAL4* strains

Previously, we classified subgroup-A JO neurons (referred to as JO-A neurons) into 13 types according to their projection patterns (Kamikouchi et al., 2006). In that study, however, only three strains that labeled the neuronal clusters innervating zone A were used, allowing us to describe a total of 49 JO-A neurons at the single-neuron level. In the present study, we increased the screening size to further evaluate the diversity of JO-A neurons.

We previously reported six *JO* strains that selectively label JO-A neurons (Kamikouchi et al., 2006; *NP* series; Figures 1B–D, Table 1). In addition to these strains, we newly identified five *GAL4* strains that labeled JO-A neurons (Figures 1B–D) by screening a database of 6650 *FlyLight* lines (Jenett et al., 2012). Together, we used 11 *GAL4* strains (hereafter referred to as *JO-A GAL4* strains) that selectively label some JO-A neurons for further analyses. The labeled subareas were variable among *JO-A GAL4* strains (Figures 1B–D), suggesting that different *GAL4* strains distinctly labeled the different populations of JO-A neurons. As the *GAL4*-expression pattern is controlled by the fragment of genomic DNA that serves as a transcriptional enhancer (*FlyLight* strains) or the genomic location of transgene insertion (*NP* strains), the different expression pattern in each strain would reflect the different expression pattern of intrinsic genes related to each enhancer fragment or genomic location.

### Neuronal types that comprise subgroup-A neurons

**Figure 2:**
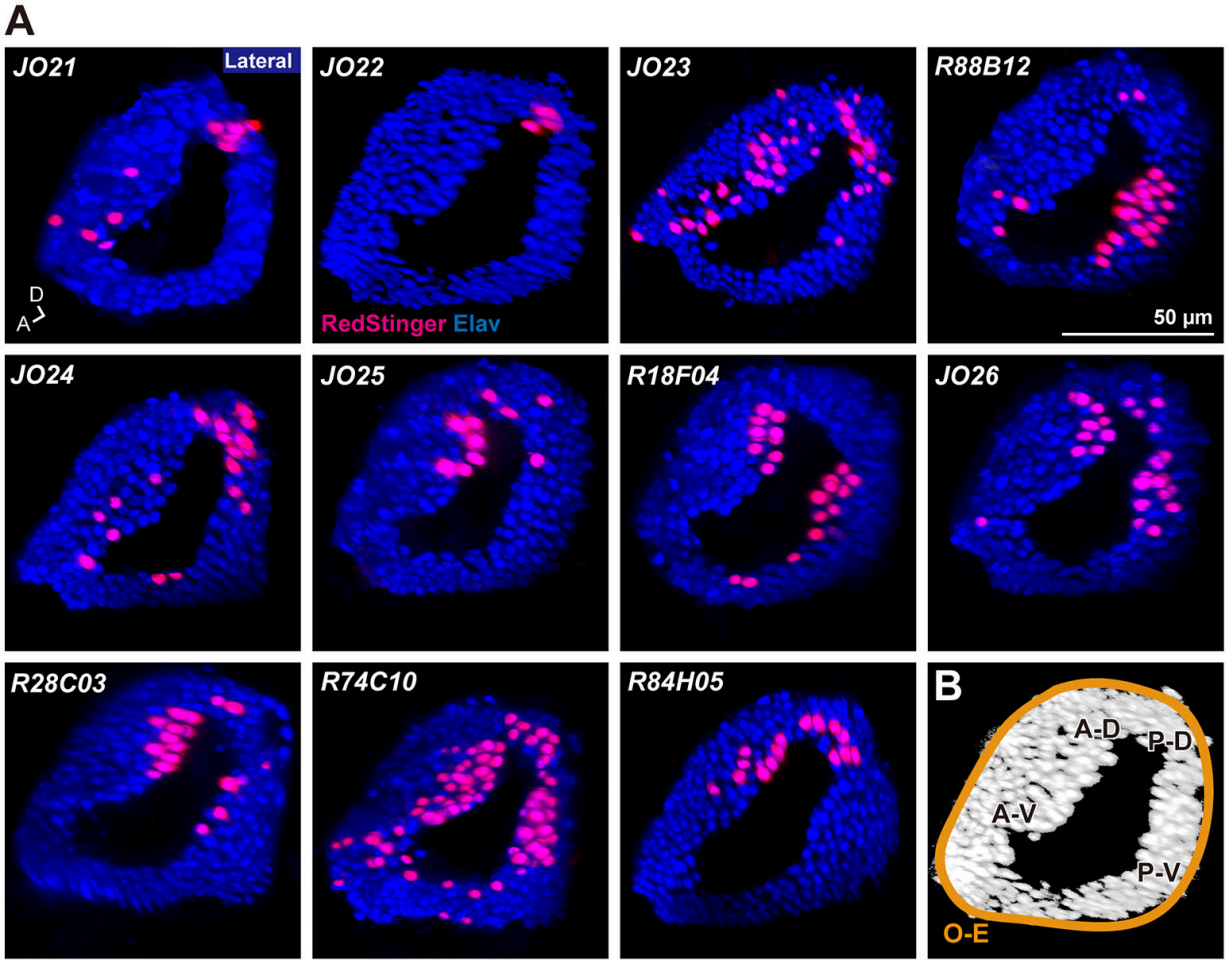
Distribution of labeled cell bodies in JO. **(A)** Distribution of the labeled cells in the somata array of JO neurons. Magenta and blue signals show the cell bodies labeled by RedStinger and anti-Elav antibody, respectively. A, anterior; D, dorsal. **(B)** Somata regions of subgroup-A neurons. A-D, anterior-dorsal; P-D, posterior-dorsal; A-V, anterior-ventral; P-V; posterior-ventral; O-E, outer-edge.

Analysis of the projection patterns revealed that each *JO-A GAL4* strain labels subsets of subareas in AMMC zone A, which varied from three (*JO21, JO22, JO23*, and *R88B12*), four (*JO24, JO25*, and *R18F04*), to all five (*JO26, R28C03, R74C10*, and *R84H05*) subareas (Figures 1B–D). Our previous report identified the somata location of JO-A neurons at the inner layer of the somata array (Kamikouchi et al., 2006). To confirm this distribution pattern, we expressed RedStinger (nuclear red fluorescent protein) using each *JO-A GAL4* strain (Figure 2). The number of labeled JO neurons in each strain ranged from 6 (*JO22*, median of three samples) to 87 (*R74C10*, median of three samples; Table 2).

**Table 2:** Subareas and cell numbers labeled in each *JO-A* strain.

**Strain**	**Labeled subareas**	**Cell number**
*JO21 (NP0799)*	AA, AP, AV2	10 ± 3 (*n* = 3)
*JO22 (NP1346)*	AA, AP, AV2	6 ± 5 (*n* = 3)
*JO23 (NP3595)*	AA, AP, AV2	39 ± 2 (*n* = 5)
*R88B12*	AA, AP, AV2	21 ± 4 (*n* = 3)
*JO24 (NP1017)*	AA, AP, AV1, AV2	12 ± 5 (*n* = 3)
*JO25 (NP5021)*	AA, AP, AV2, AD	12 ± 3 (*n* = 3)
*R18F04*	AA, AP, AV2, AD	22 ± 1 (*n* = 3)
*JO26 (NP1109)*	AA, AP, AV1, AV2, AD	26 ± 6 (*n* = 3)
*R28C03*	AA, AP, AV1, AV2, AD	15 ± 5 (*n* = 3)
*R74C10*	AA, AP, AV1, AV2, AD	87 ± 10 (*n* = 3)
*R84H05*	AA, AP, AV1, AV2, AD	19 ± 18 (*n* = 4)

*Original names of the NP-series GAL4 strain are shown in the parenthesis. Median ± standard deviation are shown*.

*R74C10* strain labeled all subareas in zone A (Figure 1D) and had the maximum number of labeled JO-A neurons (Table 2), and thus presumably covered most JO-A neurons. The labeled cell bodies in *R74C10* were distributed mainly in the inner layer of the somata array, but some of them located in the middle and outer layers (Figure 2A). Distributions of labeled cell bodies in other strains were included in the region of *R74C10* neurons (Figure 2A). We defined each location of labeled cell bodies for further analyses: anterior-dorsal (A-D), posterior-dorsal (P-D), anterior-ventral (A-V), and posterior-ventral (P-V) regions in the inner layer and outer-edge (O-E) region in the outer layer (Figure 2B).

**Figure 3:**
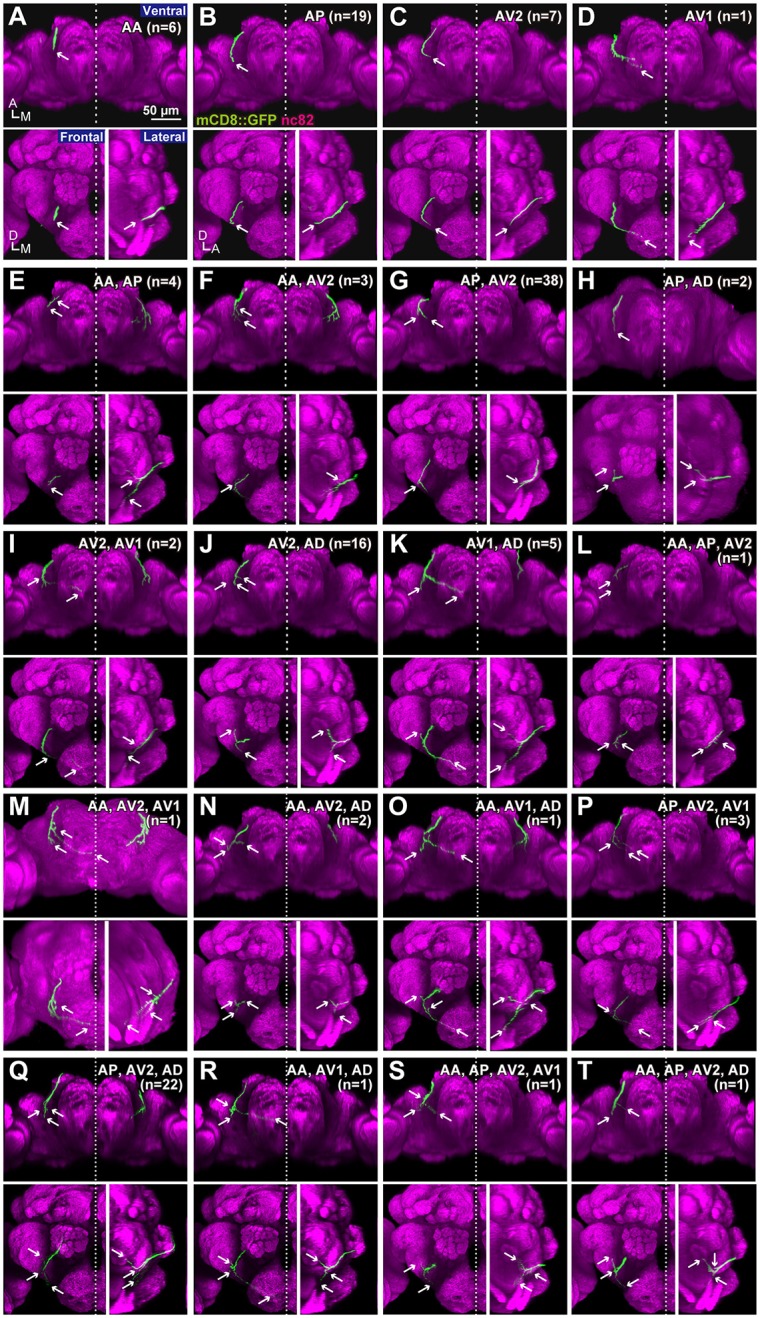
Projection patterns of JO-A neurons. Ventral, frontal, and lateral views of the brain that visualize single JO-A neurons are shown. The projection target of each neuron is shown at the upper-right of each panel. Arrows denote the projection target. Signals of cells that were not relevant to JO-A neurons were manually erased from the original images for clarity. Green and magenta signals show the neurons and neuropils labeled with mCD8::GFP and nc82 antibody, respectively. All but **(H,M)** were registered to a template brain **(A–G**, **I–L**, **N–T)**. A, anterior; D, dorsal; M, medial.

To test whether the combination of the projecting subareas of JO neurons was correlated with the somata location, we visualized single JO-A neurons using the “heat-shock FLP-out” technique, which restricts reporter expression to one or only a few GAL4-expressing cells (Basler and Struhl, 1994; Wong et al., 2002; Kamikouchi et al., 2006; Matsuo et al., 2016). To include as many neurons as possible, we also used *JO15* strain, which labeled JO-A and JO-B neurons (Kamikouchi et al., 2006), for this single-cell analysis. All strains yielded samples in which we successfully identified the cell bodies and axonal trajectories of single JO-A neurons (Table 1). We analyzed 136 neurons and classified them as “types” according to the combination of target subareas, which resulted in 20 types of neurons (7 novel types and 13 known types; Table 3, Figure 3). None of them innervated all subareas, indicating that JO-A neurons transmit signals to particular subsets of subareas of zone A.

**Table 3:** Single subgroup-A neurons obtained in each *JO-A* strain.

**Subareas**	***JO15***	***JO21***	***JO22***	***JO23***	***JO24***	***JO25***	***JO26***	***R18F04***	***R28C03***	***R74C10***	***R84H05***	***R88B12***	**Total**
AA	1			2		1					1	1	6
AP	2			3	1	2	3	1		2		5	19
AV1				1									1
AV2		1	1	2	1						2		7
AA, AP	1							1		1		1	4
AA, AV2				1	1						1		3
AP, AV2		9	6	8	7	1		1			5	1	38
AP, AD						1					1		2
AV1, AV2			1								1		2
AV1, AD	3				2								5
AV2, AD	3					9		2		1	1		16
AA, AP, AV2												1	1
AA, AV1, AV2										1			1
AA, AV1, AD	1												1
AA, AV2, AD	1					1							2
AP, AV1, AV2		1	1		1								3
AP, AV1, AD									1				1
AP, AV2, AD	2			1		3			15		1		22
AA, AP, AV1, AV2										1			1
AA, AP, AV2, AD						1							1
Total	14	11	9	18	13	19	3	5	16	6	13	9	136

**Figure 4:**
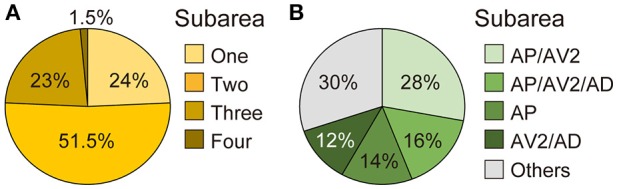
Projection target of single JO-A neurons. **(A)** Percentage of single neurons that innervate one, two, three, or four subareas. **(B)** Major types of JO-A neurons.

Many neurons (33 of 136 neurons) projected exclusively to a single subarea (Figures 3A–D, 4A). The most prominent type among them was that projecting only to subarea AP (19 of 136 neurons; hereafter referred to as JO-AP neurons; Figure 3B, Table 3). Subarea AD, on the other hand, did not receive projections from these subarea-specific neurons. Other types of neurons had two or more target subareas (Figures 3E–T, 4A). Indeed, most JO-A neurons (70 of 136 neurons) innervated two subareas (Figures 3E–K, 4A, Table 3). Among them, neurons innervating subareas AP and AV2 were the most prominent type, covering 28% of the analyzed neurons (38 of 136 neurons; hereafter referred to as JO-AP/AV2 neurons; Figures 3G, 4B). This finding is consistent with our previous report (Kamikouchi et al., 2006) and confirms the close relationship between these two subareas.

**Figure 5:**
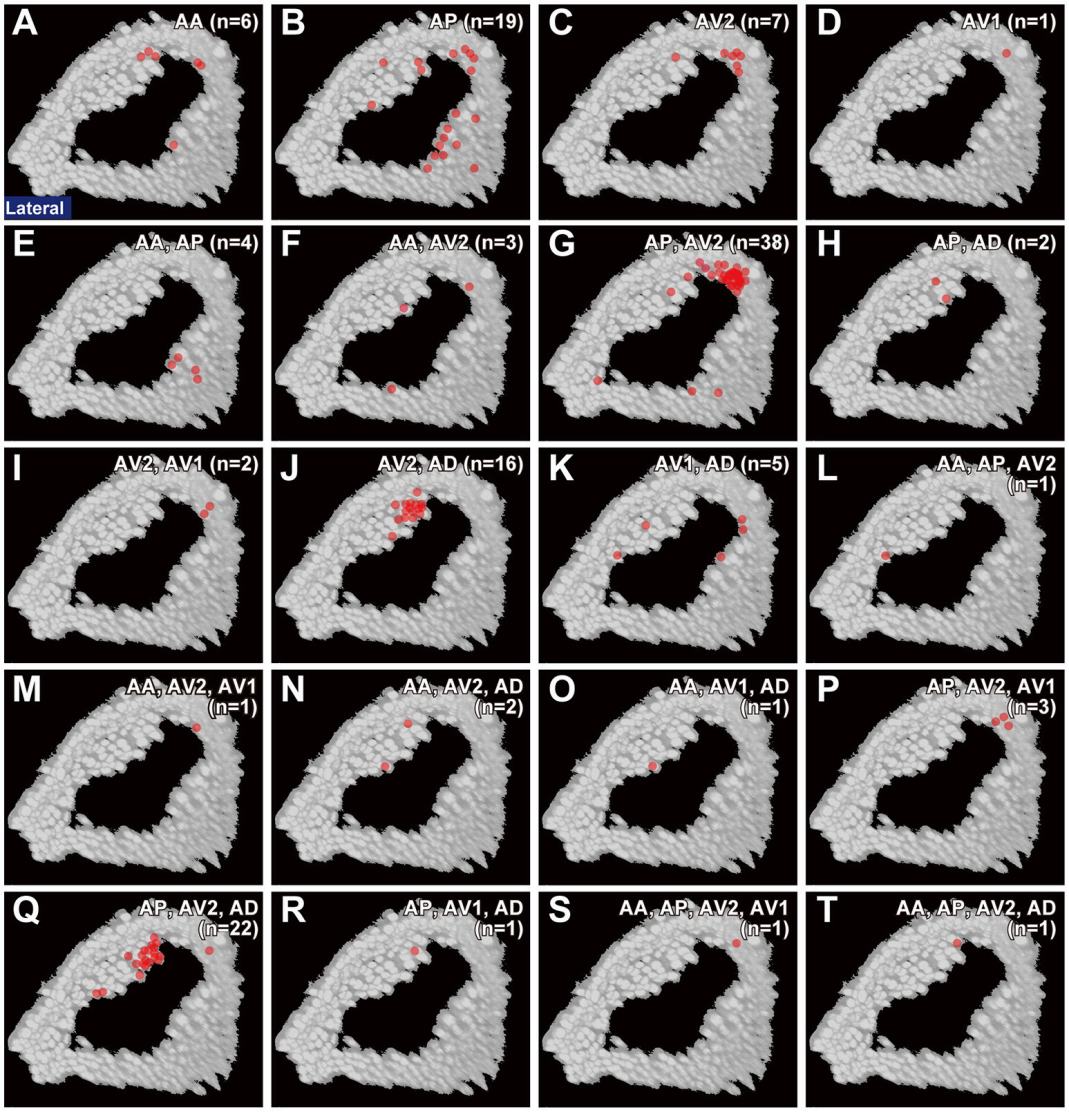
Distribution of cell bodies. Red dots in each panel indicates the location of cell bodies of JO-A neurons that project to a single subarea **(A–D)** and two or more subareas **(E–T)** in the AMMC zone A. Target subareas are shown at the upper-right of each panel. Number of samples analyzed at the single-neuron level is shown in parenthesis.

To clarify whether the cell bodies of a particular type that sends axons to the same combination of subareas are clustered together, we made a correlation map between the cell body location in the antenna and the projection pattern in the brain (Figure 5). The cell bodies of JO-AP/AV2 neurons were mostly located at the P-D region, whereas those of JO-AV2/AD neurons and JO-AP/AV2/AD neurons were mostly located at the A-D region (Figures 5G,J,Q). In contrast to those cluster-pattern neurons, the cell bodies of JO-AP neurons were scattered across all regions of the inner layer (Figure 5B). The numbers of identified cell bodies of other types of neurons were small (*n* < 10), which prevented us from identifying the distribution pattern of those cell bodies. These results indicate that whereas cell bodies of a certain type of JO-A neurons, like JO-AP/AV2 neurons, are clustered, others, like JO-AP neurons, are broadly distributed in JO.

**Figure 6:**
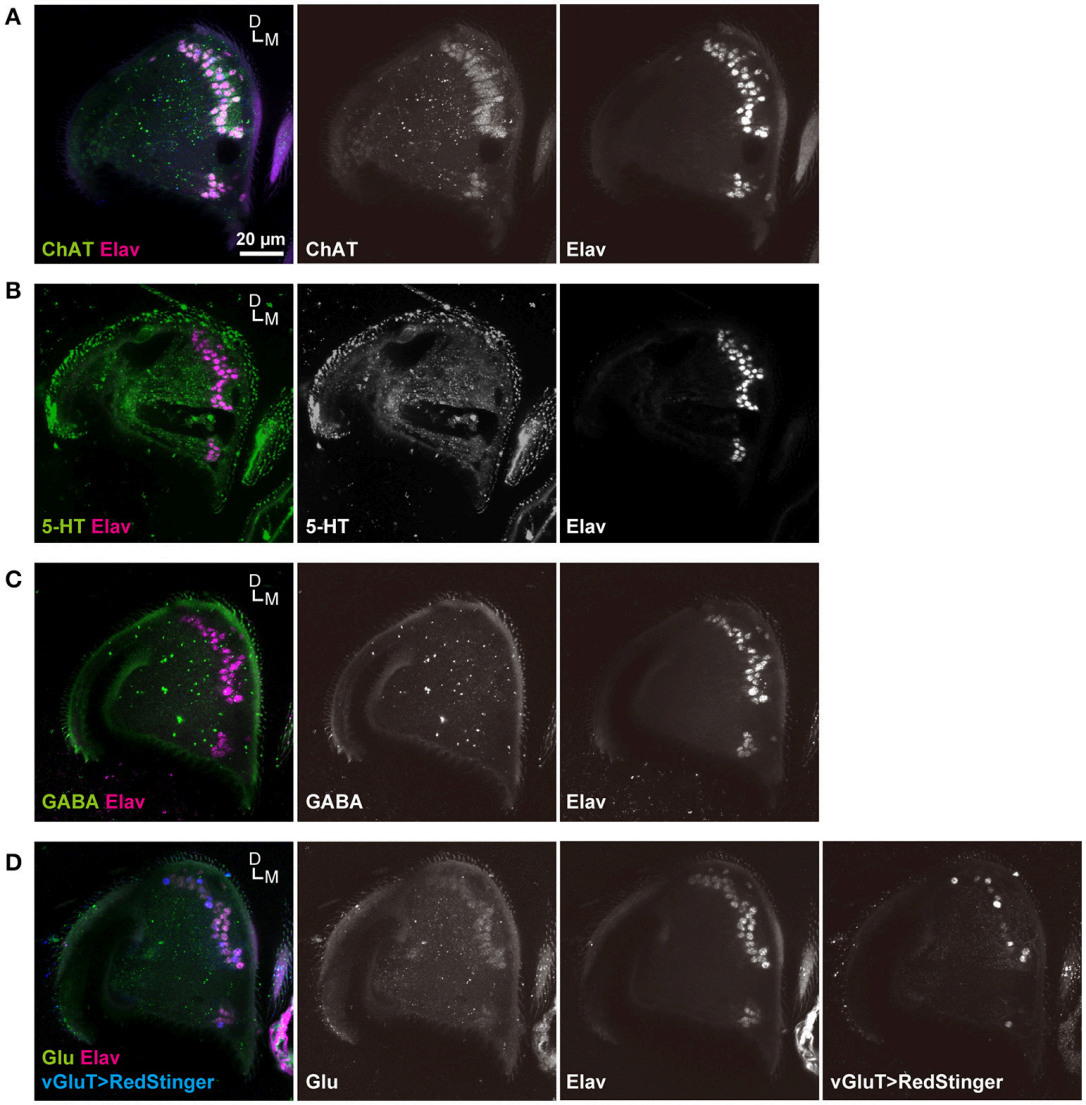
Neurotransmitter of JO neurons. JO neurons labeled with anti-ChAT **(A)**, anti-serotonin (5-HT) **(B)**, anti-GABA **(C)**, and anti-glutamate (Glu) **(D)** antibodies (green signals). The nuclei of all JO neurons were labeled with anti-ELAV antibody (magenta signals). vGluT positive neurons were labeled by a combination of *vGluT-GAL4* driver and *UAS-RedStinger* marker strains (blue signals) **(D)**. D, dorsal; M, medial.

Sensory neurons of insects typically use acetylcholine as a major neurotransmitter (Sanes and Hildebrand, 1976; Bicker, 1999; Yasuyama and Salvaterra, 1999). A few JO neurons in American cockroaches, however, use other neurotransmitters, such as serotonin (Watanabe et al., 2014). We confirmed that anti-ChAT antibodies labeled most (if not all) JO neurons (Figure 6A). On the other hand, anti-serotonin (5-hydroxytryptamine) and anti-GABA antibodies did not label these neurons (Figures 6B,C). However, anti-glutamate antibody weakly labeled the cell bodies of JO neurons (Figure 6D). Moreover, *vGluT-GAL4*, which labels most of the glutamatergic neurons (Mahr and Aberle, 2006), labeled many but not all JO neurons (Figure 6D). Together, these results indicate that most JO neurons are cholinergic, and a part of them may be glutamatergic.

### Response properties of subgroup-A neurons

In previous studies, we reported that JO-A neurons respond to acoustic stimuli in a high frequency range (>100 Hz; Kamikouchi et al., 2009; Yorozu et al., 2009; Matsuo et al., 2014). As the response properties of JO-A neurons were collectively observed in these previous studies, however, it has remained unknown whether all of these anatomically diverse types of JO-A neurons share the same response properties. To reveal the possible physiologic heterogeneity between these neurons, we analyzed the activity pattern of subsets of JO-A neurons in response to various patterns of antennal vibrations. We used *R18F04, R28C03, R74C10, R84H05*, and *R88B12* as *GAL4* drivers to induce the expression of GCaMP6m (Chen et al., 2013) as they labeled few other neurons in the brain, allowing us to selectively visualize the Ca^2+^ response of the axons of JO-A neurons. To analyze the response properties of all types of JO-A neurons, we also observed the Ca^2+^ response of *F-GAL4* strain, which labels most JO neurons (Kim et al., 2003).

**Figure 7:**
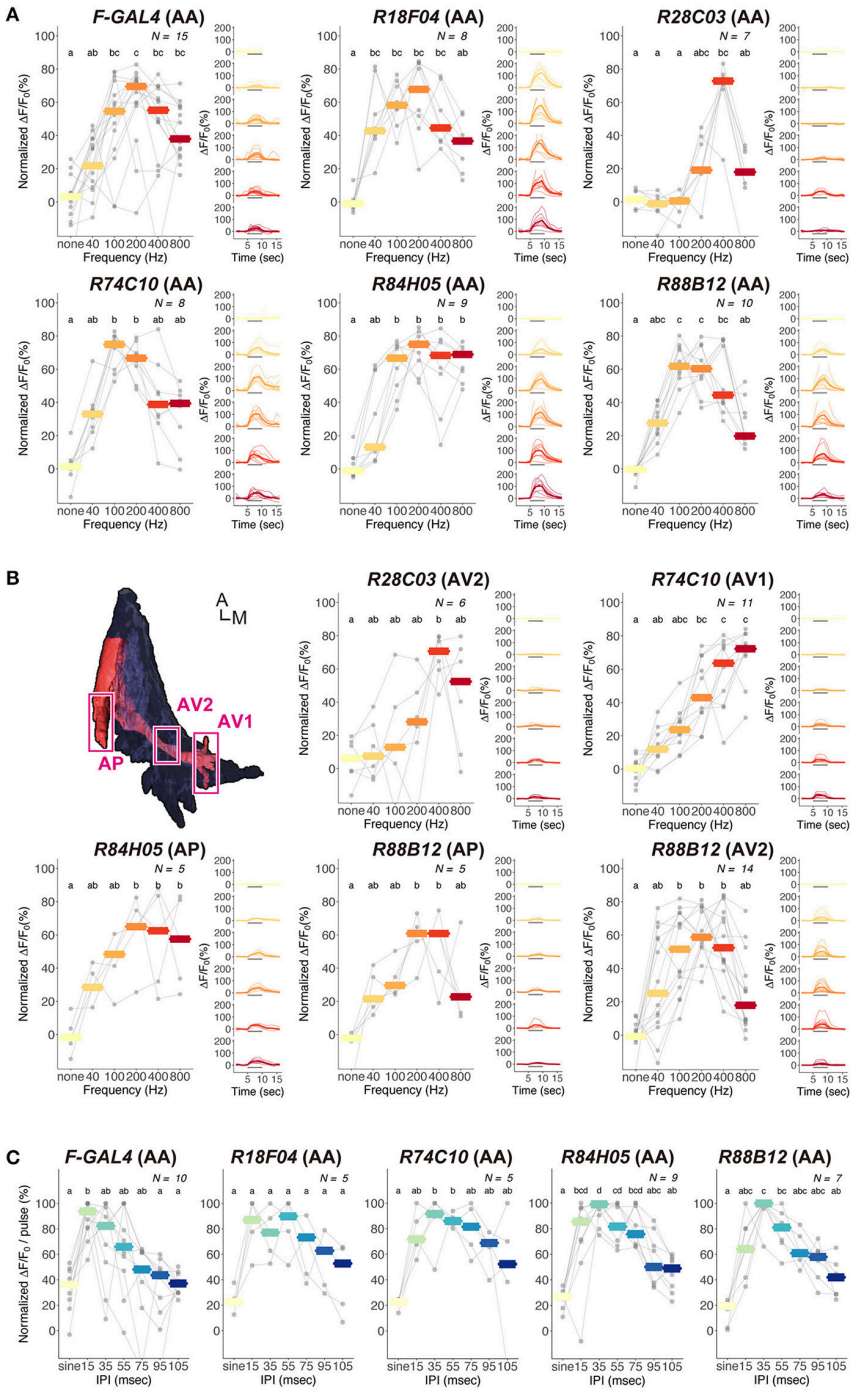
Ca^2+^ responses of JO-A neurons to antennal vibrations. **(A,B)** Ca^2+^ responses of JO-A neurons to sinusoidal vibrations in subarea AA **(A)** and other subareas **(B)**. Analyzed subarea is shown in parenthesis. Normalized (Left panel) and absolute (Right panel) values of ΔF/F_0_ are shown. (Left) Each gray point indicates the mean of fluorescent changes during vibration stimulus in each individual. Responses observed in the same individuals are connected with gray lines across different frequencies. Crossbars indicate the median response of all samples to each frequency. (Right) Time-courses of fluorescent changes are shown. Thin lines indicate the fluorescent changes in each individual, and thick lines indicate the average of the changes. Gray horizontal bars indicate the stimulus periods (5–10 s). The color codes are the same as Left. Top left panel in **(B)** indicates the region of interest analyzed in each subarea (ventral view). Different letters on the top indicate statistical significance in each *GAL4* strain (*p* < 0.05). A, anterior; M, medial. **(C)** Ca^2+^ responses of JO-A neurons to pulse-song like vibrations in subarea AA. The code for gray point, gray lines, and crossbars is the same as **(A)**. The frequency of sinusoidal stimulus (sine) is 167 Hz. Different letters on the top indicate statistical significance in each *GAL4* strains (*p* < 0.05).

FLP-out analysis revealed that each *JO-A* strain labeled distinct but overlapping combinations of JO-A neuronal types (Table 3). Because the axonal trajectory of all types of JO-A neurons is in subarea AA, we first monitored the Ca^2+^ response in subarea AA to determine the response pattern of JO-A neurons labeled with each *GAL4* strain. Although all examined GAL4-positive neurons responded to sinusoidal vibrations, the selectivity for the vibration frequency statistically varied among subsets (*p* = 0.002; Figure 7A, Table 4). *R74C10* neurons responded to vibrations of a broad frequency range (40–800 Hz), with the highest response to the middle range frequencies (100 and 200 Hz) and a decreased response to low and high frequencies (40, 400, and 800 Hz; Figure 7A, Table 5). This response property was similar to that of all JO-A neurons (*F-GAL4* neurons measured in the subarea AA), consistent with the anatomic findings indicating that *R74C10* labeled most JO-A neurons. The response properties of *R18F04* and *R88B12* neurons were maximal between 100 and 200 Hz, as observed in *R74C10* and *F-GAL4* neurons (Figure 7A, Table 5). In contrast, the characteristic frequencies of *R84H05* neurons and *R28C03* neurons differed from those of *R74C10* and *R88B12* neurons. *R84H05* neurons, 38% of which are JO-AP/AV2 neurons (Table 3), had a broad frequency spectrum with a high Ca^2+^ response between 100 and 800 Hz (Figure 7A, Table 5). *R28C03* neurons, on the other hand, had a narrow frequency spectrum with a strong preference for 400-Hz vibrations (Figure 7A, Table 5). Interestingly, the anatomy of *R28C03* neurons was extremely homogeneous; 94% were AP/AV2/AD neurons (Table 3). This observation raises the possibility that each type of JO-A neuron would have a sharp frequency characteristic. Together, these results indicate that the anatomically diversified JO-A neurons have a heterogeneous frequency response to antennal vibrations.

**Table 4:** Statistical comparison of Ca^2+^ response property among *GAL4* strains, subareas, and stimuli.

**Data**		**Df**	**Sum Sq**	**Mean Sq**	***F*-value**	***p*-value**
*F-GAL4, R18F04, R28C03, R74C10, R84H05, R88B12* (AA) for pure tones	*GAL4* strain	5	276,639	55,328	6.46	4.41E-08^***^
	Hz	1	139,198	139,198	16.26	1.03E-04^***^
	Interaction	5	93,049	18,610	2.17	0.002^***^
	Residuals	330	2,824,559	8,559		
*R28C03* (AA and AV2) for pure tones	Subarea	1	1,544	1,544	3.71	0.083
	Hz	1	7,233	7,233	17.40	1.75E-04^***^
	Interaction	1	0	0	0.00	1.000
	Residuals	74	30,762	416		
*R74C10* (AA and AV1) for pure tones	Subarea	1	1,258	1,258	1.75	0.283
	Hz	1	26,475	26,475	36.83	8.53E-07^***^
	Interaction	1	16,635	16,635	23.14	9.54E-05^***^
	Residuals	110	79,084	719		
*R84H05* (AA and AP) for pure tones	Subarea	1	529	529	1.08	0.346
	Hz	1	9,802	9,802	20.08	4.93E-05^***^
	Interaction	1	9	9	0.02	0.902
	Residuals	80	39,045	488		
*R88B12* (AA, AV2 and AP) for pure tones	Subarea	2	2,782	1,391	0.54	0.295
	Hz	1	4,359	4,359	1.70	0.190
	Interaction	2	1,221	611	0.24	0.488
	Residuals	168	430,625	2,563		
*F-GAL4, R18F04, R74C10, R84H05, R88B12* (AA) for sine and pulse songs	*GAL4* strain	4	12,731	3,183	1.05	0.111
	Factor (song)	6	526,376	87,729	28.98	0^***^
	Interaction	24	47,024	1,959	0.65	0.002^***^
	Residuals	210	635,789	3,028		

For statistical analysis, Scheirer-Ray-Hare test were performed. Asterisks indicate statistical significance (

*** *p < 0.001). Df, degree of freedom; Sq, square*.

**Table 5:** Statistical comparison of Ca^2+^ responses among sinusoidal vibrations.

***GAL4* strain**	**Statistical method**	**Pairwise**	**maxT**	***p*-value**
*F-GAL4*	Friedman test	–	5.172	6.581.E-06^***^
	*Post-hoc* test	40–0	–	0.687
		100–0	–	0.001^***^
		200–0	–	3.286.E-06^***^
		400–0	–	0.001^**^
		800–0	–	0.030
		100–40	–	0.089
		200–40	–	0.003^*^
		400–40	–	0.089
		800–40	–	0.624
		200–100	–	0.892
		400–100	–	1.000
		800–100	–	0.892
		400–200	–	0.892
		800–200	–	0.263
		800–400	–	0.892
*R18F04*	Friedman test	–	4.009	8.845.E-04^***^
	*Post-hoc* test	40–0	–	0.026
		100–0	–	0.001^**^
		200–0	–	0.001^**^
		400–0	–	0.039^*^
		800–0	–	0.765
		100–40	–	0.967
		200–40	–	0.937
		400–40	–	1.000
		800–40	–	0.507
		200–100	–	1.000
		400–100	–	0.937
		800–100	–	0.113
		400–200	–	0.894
		800–200	–	0.081
		800–400	–	0.596
*R28C03*	Friedman test	–	3.714	0.003^***^
	*Post-hoc* test	40–0	–	0.026
		100–0	–	0.001^**^
		200–0	–	0.001^**^
		400–0	–	0.039^*^
		800–0	–	0.765
		100–40	–	0.967
		200–40	–	0.937
		400–40	–	1.000
		800–40	–	0.507
		200–100	–	1.000
		400–100	–	0.937
		800–100	–	0.113
		400–200	–	0.894
		800–200	–	0.081
		800–400	–	0.596
*R74C10*	Friedman test	–	4.410	1.695.E-04
	*Post-hoc* test	40–0	–	0.596
		100–0	–	1.540.E-04^***^
		200–0	–	2.725.E-04^***^
		400–0	–	0.154
		800–0	–	0.507
		100–40	–	0.057
		200–40	–	0.081
		400–40	–	0.967
		800–40	–	1.000
		200–100	–	1.000
		400–100	–	0.340
		800–100	–	0.081
		400–200	–	0.420
		800–200	–	0.113
		800–400	–	0.985
*R84H05*	Friedman test	–	4.410	1.388.E-04^***^
	*Post-hoc* test	40–0	–	0.573
		100–0	–	0.009^**^
		200–0	–	1.495.E-04^***^
		400–0	–	0.009^**^
		800–0	–	0.009^**^
		100–40	–	0.489
		200–40	–	0.062
		400–40	–	0.489
		800–40	–	0.489
		200–100	–	0.916
		400–100	–	1.000
		800–100	–	1.000
		400–200	–	0.916
		800–200	–	0.916
		800–400	–	1.000
*R88B12*	Friedman test	–	4.781	4.822.E-05^***^
	*Post-hoc* test	40–0	–	0.159
		100–0	–	2.738.E-05^***^
		200–0	–	2.738.E-05^***^
		400–0	–	0.001^***^
		800–0	–	0.394
		100–40	–	0.159
		200–40	–	0.159
		400–40	–	0.550
		800–40	–	0.997
		200–100	–	1.000
		400–100	–	0.980
		800–100	–	0.047
		400–200	–	0.980
		800–200	–	0.047^*^
		800–400	–	0.261

For statistical analysis, Friedman test followed by post-hoc Wilcoxon-Nemenyi-McDonalds-Thompson test were performed. Asterisks indicate statistical significance (

*** *p < 0.001*,

** *p < 0.01*,

* *p < 0.05)*.

Subareas other than subarea AA contain axons of specific types of JO-A neurons that innervate each subarea. We next investigated whether the frequency selectivity observed in subarea AA was maintained in other subareas. In most cases, other subareas had a similar frequency preference as subarea AA in each *GAL4* strain; subarea AV2 of *R28C03* neurons, subarea AP of *R84H05* neurons, and subareas AP and AV2 of *R88B12* neurons had a response property similar to the responses measured in subarea AA of the corresponding strain (*p* = 1.000 in *R28C03, p* = 0.902 in *R84H05, p* = 0.488 in *R88B12*; Figure 7B, Table 4). Single neuron analysis revealed that subarea AP of *R84H05* neurons was dominated by JO-AP/AV2 neurons. On the other hand, subarea AP of *R88B12* neurons was predominantly occupied by JO-AP neurons. These two neuronal types likely have a similar frequency spectrum at the low and middle-range frequencies (40–400 Hz), but differed at the high frequency range (800 Hz). Subarea AP thus receives signals from, at least, two types of JO-A neurons, each of which has a distinct response property at the high-frequency range.

In *R74C10* neurons, the response properties of subarea AV1 were strikingly different from those of subarea AA; as the vibration frequency increased, subarea AV1 of *R74C10* showed higher Ca^2+^ responses (*p* = 9.54E-05; Figure 7B, Table 4). Single-neuron analysis revealed that subarea AV1 of *R74C10* neurons contained at least two neuronal types (JO-AA/AV1/AV2 and JO-AA/AP/AV1/AV2 neurons; Table 3). The Ca^2+^ response of subarea AV1 would thus represent the properties of these specific types of JO-A neurons, whose frequency selectivity was distinct from that observed in subarea AA of *R74C10*.

Both male and female *D. melanogaster* exhibit selective behavioral responses to the pulse song with a species-specific IPI (about 35 ms in *D. melanogaster*; Ewing and Bennet-Clark, 1968). To reveal whether the JO-A neurons show a preference to the species-specific IPI, we further investigated the Ca^2+^-response selectivity of JO-A neurons to antennal vibrations that mimicked artificial pulse songs with various IPIs (Figure 7C). To evaluate the response, we fixed the number of stimulus (20 pulses for 15–105-ms IPIs and 120 ms stimulus for 167 Hz sinusoidal vibrations; see Section Materials and Methods for details). When we analyzed the response properties of all types of JO-A neurons (*F-GAL4*), a pulse song with a shorter IPI induced higher Ca^2+^ responses, whereas the responses decreased as the IPI became longer (Figure 7C, Table 6). Sinusoidal vibrations induced a low response, similar to a pulse song with a long IPI. We compared the response pattern to these IPI series between *JO-A GAL4*s, each of which labeled specific subsets of JO-A neurons; the IPI-response properties were statistically different among these subsets (*p* = 0.002; Table 4). Interestingly, *R84H05 and R88B12* neurons showed a preference that peaked at the 35-ms IPI. This response property is consistent with the species-specific IPI, which induced higher behavioral response than the 15-ms IPI song in *D. melanogaster* (Yoon et al., 2013). This result raises the possibility that JO-A neurons are involved in processing the courtship song of flies.

**Table 6:** Statistical comparison of Ca^2+^ response among various IPIs.

***GAL4* strain**	**Statistical method**	**Pairwise**	**maxT**	***p*-value**
*F-GAL4*	Friedman test	–	3.933	0.002^**^
	*Post-hoc* test	IPI15-sine	–	0.003^**^
		IPI35-sine	–	0.100
		IPI55-sine	–	0.310
		IPI75-sine	–	0.968
		IPI95-sine	–	1.000
		IPI105-sine	–	1.000
		IPI35-IPI15	–	0.916
		IPI55-IPI15	–	0.645
		IPI75-IPI15	–	0.058
		IPI95-IPI15	–	0.002^**^
		IPI105-IPI15	–	0.002^**^
		IPI55-IPI35	–	0.999
		IPI75-IPI35	–	0.575
		IPI95-IPI35	–	0.076
		IPI105-IPI35	–	0.076
		IPI75-IPI55	–	0.878
		IPI95-IPI55	–	0.255
		IPI105-IPI55	–	0.255
		IPI95-IPI75	–	0.946
		IPI105-IPI75	–	0.946
		IPI105-IPI95	–	1.000
*R18F04*	Friedman test	–	2.928	0.053
*R74C10*	Friedman test	–	3.074	0.034^*^
	*Post-hoc* test	IPI15-sine	–	0.163
		IPI35-sine	–	0.034^*^
		IPI55-sine	–	0.034^*^
		IPI75-sine	–	0.115
		IPI95-sine	–	0.676
		IPI105-sine	–	0.766
		IPI35-IPI15	–	0.997
		IPI55-IPI15	–	0.997
		IPI75-IPI15	–	1.000
		IPI95-IPI15	–	0.976
		IPI105-IPI15	–	0.948
		IPI55-IPI35	–	1.000
		IPI75-IPI35	–	0.999
		IPI95-IPI35	–	0.766
		IPI105-IPI35	–	0.676
		IPI75-IPI55	–	0.999
		IPI95-IPI55	–	0.766
		IPI105-IPI55	–	0.676
		IPI95-IPI75	–	0.948
		IPI105-IPI75	–	0.905
		IPI105-IPI95	–	1.000
*R84H05*	Friedman test	–	5.019	8.642.E-06^***^
	*Post-hoc* test	IPI15-sine	–	0.026
		IPI35-sine	–	6.644.E-06^***^
		IPI55-sine	–	0.001^**^
		IPI75-sine	–	0.004^**^
		IPI95-sine	–	0.728
		IPI105-sine	–	0.977
		IPI35-IPI15	–	0.511
		IPI55-IPI15	–	0.977
		IPI75-IPI15	–	0.998
		IPI95-IPI15	–	0.658
		IPI105-IPI15	–	0.248
		IPI55-IPI35	–	0.958
		IPI75-IPI35	–	0.848
		IPI95-IPI35	–	0.009^**^
		IPI105-IPI35	–	0.007^**^
		IPI75-IPI55	–	1.000
		IPI95-IPI55	–	0.156
		IPI105-IPI55	–	0.026^*^
		IPI95-IPI75	–	0.305
		IPI105-IPI75	–	0.068
		IPI105-IPI95	–	0.995
*R88B12*	Friedman test	–	4.949	2.049.E-05^***^
	*Post-hoc* test	IPI15-sine	–	0.067
		IPI35-sine	–	1.676.E-05^***^
		IPI55-sine	–	0.002^**^
		IPI75-sine	–	0.067
		IPI95-sine	–	0.281
		IPI105-sine	–	0.823
		IPI35-IPI15	–	0.351
		IPI55-IPI15	–	0.924
		IPI75-IPI15	–	1.000
		IPI95-IPI15	–	0.996
		IPI105-IPI15	–	0.754
		IPI55-IPI35	–	0.956
		IPI75-IPI35	–	0.351
		IPI95-IPI35	–	0.093
		IPI105-IPI35	–	0.006^**^
		IPI75-IPI55	–	0.924
		IPI95-IPI55	–	0.594
		IPI105-IPI55	–	0.126
		IPI95-IPI75	–	0.996
		IPI105-IPI75	–	0.754
		IPI105-IPI95	–	0.978

For statistical analysis, Friedman test followed by post-hoc Wilcoxon-Nemenyi-McDonalds-Thompson test were performed. Asterisks indicate statistical significance (

*** *p < 0.001*,

** *p < 0.01*,

* *p < 0.05)*.

### Are JO-A neurons important for auditory behavior?

**Figure 8:**
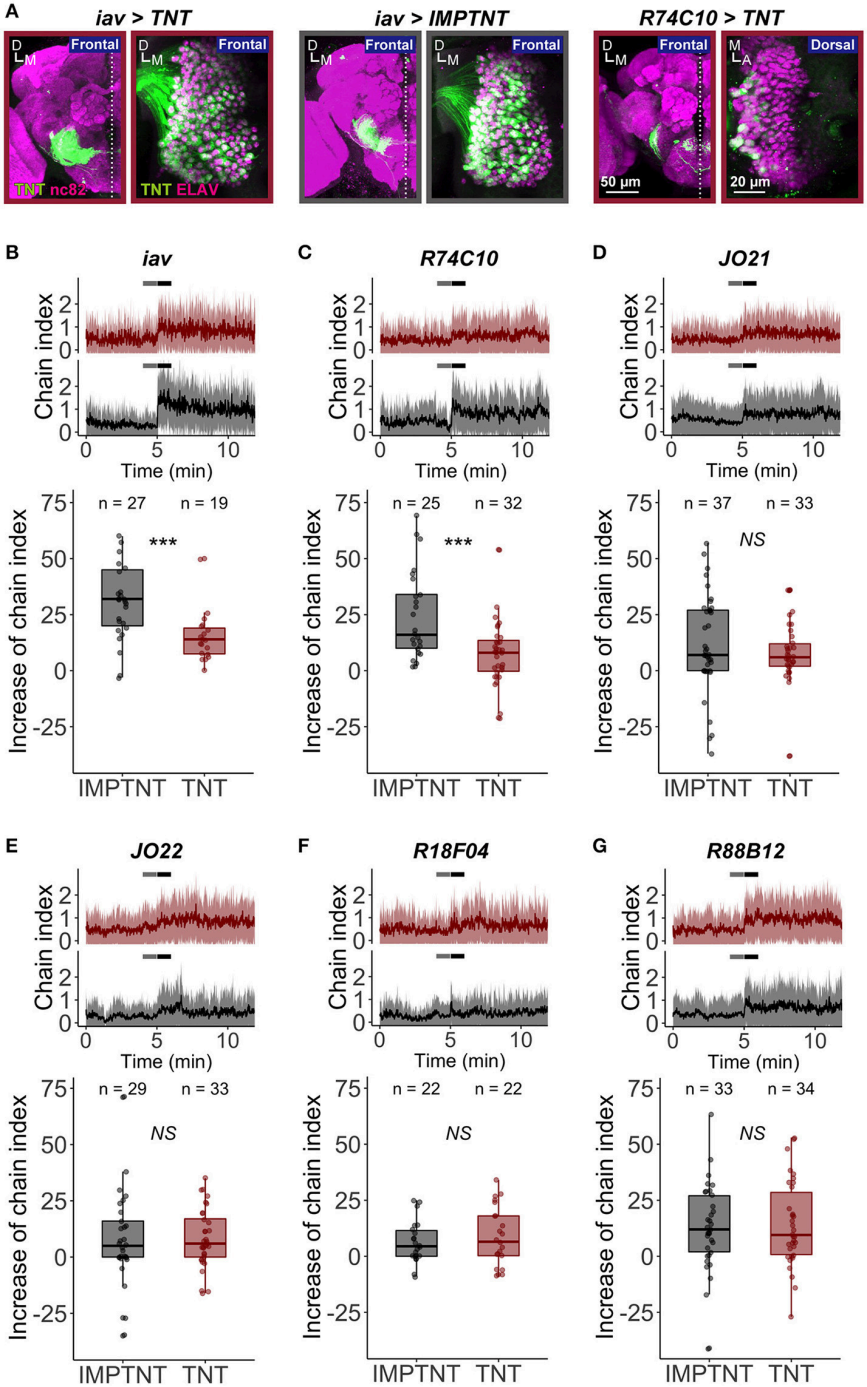
The role of JO-A neurons in the sound-evoked behavior. **(A)** Tetanus toxin (TNT) expression pattern of *iav*>*TNT, iav*>*IMPTNT*, and *R74C10*>*TNT*. TNT and IMPTNT expression was labeled with anti-TNT antibodies (green signals). Brain (Left) and Johnston's organ in the antennal second segment (Right) are shown. Neuropils in brains and nuclei of JO neurons in antennae were labeled with nc82 antibodies and ant-ELAV antibodies, respectively (magenta signals). A, anterior; D, dorsal; M, medial. **(B–G)** The chain index in response to an artificial pulse song when *iav* **(B)**, *R74C10* **(C)**, *JO21* **(D)**, *JO22* **(E)**, *R18F04* **(F)**, and *R88B12* neurons **(G)** are silenced, respectively. (Top) Red and gray traces show the time-course of the chain index of experimental (TNT, red) and control (IMPTNT, gray) flies, respectively. Sound playback starts at 5 min. Thick lines and shadows represent mean ± standard deviation. Time windows for two temporal phases (before and after) are indicated in gray and black horizontal lines. (Bottom) Increases from the temporal phase “before” to that of “after” are plotted. Each point indicates the increase in each experiment. Box plots show median (solid horizontal line), 50th percentile (box outline), and 90th percentile (whiskers) values.

Males of many *Drosophila* species produce a stereotyped courtship song to attract females. Playback experiments revealed that an artificial courtship song containing a species-specific IPI facilitates copulation behavior in both males and females (Ritchie et al., 1999). In a single-sex group situation, male flies show intensive homosexual courtship activities, displayed as chaining behavior, when they are exposed to a synthetic courtship song (Eberl et al., 1997; Yoon et al., 2013). To examine whether JO-A neurons are important for the behavioral response to the courtship song, we expressed TNT (Sweeney et al., 1995) to inhibit the synaptic transmission of JO-A neurons. First, we evaluated the effectiveness of TNT expression in suppressing the chaining behavior. We used *iav-GAL4* as a driver that labels most JO neurons, which was confirmed by detecting TNT (active form of TNT) and IMPTNT (inactive form of TNT) expressions in the brain and antennae (Figure 8A; Kwon et al., 2010). Although, the significant increase of chaining behavior at the sound onset was observed both in *iav*>*TNT* (TNT was expressed by *iav-GAL4* strain; *p* = 7.63E-06) and in control *iav*>*IMPTNT* (IMPTNT was expressed by *iav-GAL4* strain; *p* = 7.45E-08), the intensity of the chaining behavior in *iav*>*TNT* was less intense than that in the control group (*p* = 2.42E-04; Figure 8B, Table 7). We next inhibited the synaptic transmission of most JO-A neurons using *R74C10* strain as a driver strain (Figure 8A). The intensity of the chaining behavior in *R74C10*>*TNT* was attenuated when compared to that in the control group (*p* = 6.96E-04, Figure 8C, Table 7), although again the behavior was not completely inhibited. These results suggest that JO-A neurons would be important for the behavioral response to the courtship song.

**Table 7:** Statistics of chain index in *GAL4*>*TNT* males.

***GAL4* strain**	***UAS* strain**	**Comparison of chain indices between before and after sound stimulus (Exact Wilcoxon signed rank test)**				**Comparison of chain indices between TNT and IMPTNT (Exact Wilcoxon rank sum test)**			
		**V**	***p*-value**	**Rank of p**	**Alpha level**	**W**	***p*-value**	**Rank of p**	**Alpha level**
*iav-GAL4*	*UAS-TNT*	0	**7.63E-06**	3	0.006	415	**2.42E-04**	1	0.008
	*UAS-IMPTNT*	3	**7.45E-08**	2	0.005				
*R74C10*	*UAS-TNT*	92.5	**0.002**	8.5	0.030	606	**0.001**	2	0.012
	*UAS-IMPTNT*	0	**5.96E-08**	1	0.004				
*JO21*	*UAS-TNT*	58.5	**3.61E-05**	4	0.007	694.5	0.326	3	0.019
	*UAS-IMPTNT*	104	**0.002**	8.5	0.030				
JO22	*UAS-TNT*	92	**0.001**	7	0.017	467.5	0.880	4	0.050
	*UAS-IMPTNT*	59	**0.014**	12	0.050				
*R18F04*	*UAS-TNT*	42	**0.009**	11	0.050	214.5	0.526	5	0.033
	*UAS-IMPTNT*	23	**0.005**	10	0.050				
*R88B12*	*UAS-TNT*	57	**6.07E-05**	5	0.009	560.5	0.998	6	0.050
	*UAS-IMPTNT*	67.5	**1.80E-04**	6	0.012				

*Bold characters indicate statistical significance. Alpha levels were corrected by a modified Benjamini and Hochberg method to keep the false discovery rate below 5% (see Section Materials and Methods for details)*.

To test whether the sound-induced chaining behavior was attributed to specific subsets of JO-A neurons, we expressed TNT using other *JO-A GAL4* strains (Figures 8D–G). JO-AP/AV2 neurons, which represent the most prominent type of subgroup-A neurons, were dominantly labeled in *JO21* and *JO22* strains (82% in *JO21* and 67% in *JO22*; Table 3). Suppression of these neurons, however, did not decrease the chaining behavior; the experimental group (>*TNT*) showed an increase in the chain index to virtually the same level as that of the control group (>*IMPTNT*) after the sound onset (*p* = 0.326 in *JO21, p* = 0.880 in *JO22*; Figures 8D,E, Table 7). Moreover, both *R18F04*, which labels at least four types of neurons, and *R88B12*, which dominantly labels JO-AP neurons, led to an increase in the chain index in both the experimental and control groups (*p* = 0.526 in *R18F04, p* = 0.998 in *R88B12*; Figures 8F,G, Table 7). Together, our results suggest that JO-A neurons as a whole would be important for evoking the behavioral response to the courtship sound. It should be noted, however, that *R74C10* expression was also observed in the thoracicoabdominal ganglion, mainly in the putative sensory nerves that connect the appendages and thoracicoabdominal ganglion (FlyLight image database; http://flweb.janelia.org/cgi-bin/flew.cgi). Therefore, we cannot exclude the possibility that neurons other than JO-A neurons might play a dominant role in this behavioral attenuation. Our results also suggested that specific subsets of JO-A neurons, such as JO-AP/AV2 and JO-AP neurons, might not be necessary for the behavioral response to the courtship sound. It is also possible that the TNT did not effectively block synaptic transmission in these neurons, which may be why the flies still chained in response to the stimulus.

## Discussion

This study is the first to comprehensively reveal an organization of the sensory neurons in the fruit fly tuned to high-frequency sound, JO-A neurons, at the anatomic, physiologic, and functional levels.

### Anatomic heterogeneity of JO-A neurons

The projection patterns of the high-frequency neurons, JO-A neurons, are heterogeneous; at least 20 types of JO-A neurons exist in the fruit fly. Does this heterogeneity reflect a topographic representation of some parameters of the acoustic stimulus? Indeed, peripheral tonotopic maps are an important encoding scheme in both insects and vertebrates (Hildebrandt, 2014); the projections of primary auditory neurons are systematically arranged so that the central projection target shows a tonotopic arrangement (Römer, 1983; Nayagam et al., 2011). In most insects, however, tonotopic maps are quickly integrated to form more categorical representations (Hildebrandt, 2014). Such a fast transformation of a tonotopic map into categorical “labeled lines” is evident in the cricket and bushcricket auditory pathway (Hildebrandt, 2014). Thus, while in the mammalian auditory system, a peripheral feature map is maintained throughout many of the central processing stages, in insects a very similar peripheral map is rapidly integrated at the next processing level.

In fruit flies, three subgroups, JO-A, JO-B, and JO-D neurons, of auditory sensory neurons respond strongly to pure tones; each of these subgroups has a distinct but overlapping characteristic frequency (Kamikouchi et al., 2009; Yorozu et al., 2009; Matsuo et al., 2014). Each subgroup sends axonal projections to a distinct zone in the primary auditory center in the fly brain and thus organizes a primitive “tonotopic” map (Matsuo et al., 2014). In this map, JO-A neurons are tuned to high-frequency vibrations. Here we revealed that an anatomically homogeneous neural population, JO-AP/AV2/AD neurons labeled in *R28C03*, exhibited sharp frequency selectivity. This finding indicates that other types of JO-A neurons may also have such sharp frequency selectivity. Further, we found that a specific set of neural populations that possess distinct response properties projects to each subarea (e.g., *R84H05* and *R88B12* neurons projecting to subarea AP, and *R28C03* and *R88B12* neurons projecting to subarea AV2). These results together suggest that the fine frequency information of an acoustic stimulus, separated by subsets of JO-A neurons, could be transferred to a certain combination of subareas in zone A; each subarea might integrate the differently-filtered auditory information derived from the distinct neural populations. Heterogeneous JO-A neurons could distribute the filtered acoustic information into several distinct pathways, which possibly reflect some categorical “labeled lines” as observed in the cricket and bushcricket auditory pathway.

### Physiologic heterogeneity in JO-A neurons

The Ca^2+^-imaging analysis indicated that the frequency tuning of JO-A neurons is also heterogeneous. This indicates that the broad response selectivity of JO-A neurons described previously is attributed to the summation of distinct response properties in a heterogeneous neural population. In contrast to insect tympanal ears and mammalian cochlea, in which frequency tuning is provided by the mechanics of the sound-receiving and sound-transmitting structures, the insect antennal ear functions as a single resonant filter (Göpfert and Hennig, 2016). Because all JO neurons would experience the same mechanical frequency filtering by the antenna, the mechanism underlying the different response properties of JO-A neurons could be attributed to intrinsic acoustic tuning processes.

One well-known example of the intrinsic mechanism is electrical tuning, which is explained by the electrical resonance of each neuron (Hutcheon and Yarom, 2000). In turtles, the resonant frequencies of hair cells vary systematically along the length of the basilar papilla (Fettiplace and Fuchs, 1999). Electrical tuning, which is also observed in the hair cells of fish, frogs, alligators, and chicks, may be generated by an interaction between a voltage-gated inward Ca^2+^ current and a Ca^2+^-dependent outward K^+^ current flowing through large conductance Ca^2+^-activated K^+^ (BK) channels (Ashmore, 1983; Fuchs and Evans, 1988; Sugihara and Furukawa, 1989; Steinacker and Romero, 1992; Fettiplace and Fuchs, 1999). These currents play a key role in frequency tuning by contributing to the membrane oscillations that set the characteristic frequency at which each cell is most sensitive (Fettiplace and Fuchs, 1999). By analogy, it is possible that the different electrical tuning produced by the difference in BK channels supports the distinct frequency tuning observed in subsets of JO-A neurons in fruit flies (Göpfert and Hennig, 2016).

The kinetics of membrane oscillations can be regulated by the amount of BK channels. In concordance with previous electrophysiologic data from turtle hair cells, BK channel clusters increase as cells are sampled from the low frequency to the high frequency region of the basilar papilla in chicks, although the cluster number does not always reflect the number of channel molecules (Samaranayake et al., 2004). The structural variation in BK channels is another possible mechanism that regulates the membrane kinetics. BK channels are encoded by a single *slowpoke (slo)* gene in fruit flies, and by a homologous gene, *Slo1*, in mammals (Atkinson et al., 1991; Fettiplace and Fuchs, 1999; Salkoff et al., 2006). A large number of splice variants have been identified in both vertebrates and insects (Atkinson et al., 1991; Adelman et al., 1992; Butler et al., 1993; Lagrutta et al., 1994; Navaratnam et al., 1997; Rosenblatt et al., 1997), some of which produce channels with significantly different Ca^2+^ sensitivities and kinetics (Tseng-Crank et al., 1994; Navaratnam et al., 1997; Rosenblatt et al., 1997). In the chicken cochlea, BK channel isoforms distribute along the tonotopic gradient and exhibit variations in Ca^2+^ and voltage sensitivity, suggesting that the spatial distribution of the variants contributes to determine the tonotopic map (Rosenblatt et al., 1997). In *Drosophila*, 23 splicing variants of *slo* are reported, some of which have altered gating kinetics (Lagrutta et al., 1994). Thus, the heterogeneity of the Ca^2+^ response properties in JO-A neurons may be due to variations in the electric resonance determined by the amount and variation of BK channels expressed.

### Subgroup-A neurons for detecting the courtship song

We used sound-induced chaining behavior to evaluate the ability of flies to transmit acoustic signals from the antennal ear to the brain. When most JO neurons were silenced by TNT (*iav*>*TNT* flies), chaining behavior was attenuated but not entirely lost. There are two possible explanations for this sustained chain response. Because *iav-GAL4* does not label all the JO neurons, the first explanation is that residual JO neurons that do not express TNT send acoustic information to the brain, which then leads to the weak behavioral response. The other one is that the TNT expression does not abolish but attenuates the function of the targeted neurons in our experimental condition. In either case, TNT expression brought a significant impact to the behavioral output, allowing us to estimate the function of targeted neurons in our experiment.

When the maximum number of JO-A neurons were silenced by TNT, chaining behavior was attenuated. This is the first experimental data suggesting that JO-A neurons would contribute to the auditory behavioral response of fruit flies. This contrasts with our previous report that the suppression of subgroup A, C, and E neurons did not affect chaining behavior (Kamikouchi et al., 2009). This discrepancy can be explained by the labeling pattern of *GAL4* strains used in these two reports; *JO4* (also known as *NP6303*), which was used in the previous report, did not label all JO-A neurons. Indeed in *JO4* strain, few JO-A neurons in the A-D and P-D regions of the inner layer were labeled in the JO, and no projections to subarea AD were labeled in the brain (Kamikouchi et al., 2006). In the present study, we showed the suppression of most JO-A neurons, but not smaller subsets of JO-A neurons, decreased chaining behavior. This suggests that the whole population (or a large portion) of JO-A neurons rather than particular JO-A neurons is possibly important for auditory behavioral responses. Despite the anatomic and physiologic heterogeneity of JO-A neurons, the functional heterogeneity is likely limited so that smaller subsets can be compensated for by other subsets of JO-A neurons to elicit the behavioral response to the courtship song. There is a caveat, however, to conclude that the observed behavioral phenotypes were due to the blockage of JO-A neurons; as described in Results, *R74C10*, which was used to silence the maximum number of JO-A neurons, also labels neurons in the thoracicoabdominal ganglion. Further behavioral analysis is required to elucidate the function of JO-A neurons and their subsets in the auditory pathway that controls courtship behavior.

### Function of JO-A neurons in auditory processing

The findings of the present study suggested for the first time that JO-A neurons, which are anatomically and physiologically heterogeneous, would contribute to auditory responses in the fruit fly. If this is the case, what roles do JO-A neurons play in auditory processing? Observations of the sound-evoked behavior and neural activity in the auditory pathway of crickets led to a concept of serially arranged filtering mechanisms to recognize species-specific acoustic signals (Hedwig, 2016). From this point of view, JO-A neurons could function as a filter that passes high-frequency (>100 Hz) vibrations. Based on the finding that the courtship activity of flies is increased by exposure to the pulse song, which includes a wide range of frequency components, but not affected dramatically by the narrowband sine song, high-frequency pass filtering of JO-A neurons is potentially important for detecting the species-specific courtship song. Further, the finding that JO-B neurons, which prefer low-frequency (<100 Hz) vibrations, and their downstream neurons are also important for the behavioral response to the pulse song of flies (Kamikouchi et al., 2009; Zhou et al., 2015) suggests that information integration of low (<100 Hz) and high frequency (>100 Hz) vibrations is required for pulse-song detection. Interestingly, several subsets of JO-A neurons strongly respond to an artificial pulse song that carries a conspecific 35-ms IPI. These neurons could selectively pass the conspecific pulse song to the downstream neurons.

The heterogeneity of the response properties of JO-A neurons revealed in this study suggests that morphologically distinct JO-A neurons send outputs to the downstream neurons through filters that have overlapping, but distinct, properties. A large-scale analysis of secondary auditory neurons in the brain identified 19 types of interneurons downstream of JO-A neurons, all of which innervate specific subarea(s) in zone A (Matsuo et al., 2016). Given that one subarea receives projections from several types of JO-A neurons, the differently filtered auditory information could be integrated in these downstream neurons. This complicated organization between the auditory primary and secondary neurons may contribute to the auditory processing that filters species-specific sound stimuli.

What kind of information processing is performed in the auditory pathway of the fruit fly? Pulse songs that carry conspecific IPIs effectively increase mating behavior in both male and female flies compared to heterospecific songs (Ritchie et al., 1999), suggesting that flies have the ability to discriminate the conspecific IPI. In addition to the courtship song during mating behavior, agonistic sounds are reportedly generated by flies during aggressive behavior (Jonsson et al., 2011). Interestingly, agonistic songs, which exclusively comprise pulses with an IPI twice as long and more variable than that of courtship songs, initially induce chaining behavior, but it is rapidly attenuated (Yoon et al., 2013). This observation suggests that IPI variations are evaluated in the auditory pathway of flies. One possible mechanism underlying such evaluation is a system that compares the output of several distinct pathways. As shown in this study, distinct subsets of JO-A neurons exhibit different IPI preferences. This heterogeneity might contribute to compute the differences and variations of IPIs in the downstream auditory pathway. Further behavioral analysis is required to validate this speculation.

## Ethics statement

The research performed in this study on the fruit fly, *Drosophila melanogaster*, did not require approval by an ethics committee.

## Author contributions

YI and AK desinged the study. YI, NO, MN, HK, and AK performed the experiments and analyzed the data. YI and AK wrote the paper. All the authors read and approved the final manuscript.

### Conflict of interest statement

The authors declare that the research was conducted in the absence of any commercial or financial relationships that could be construed as a potential conflict of interest.

## Abbreviations

IPI: Interpulse interval
JO: Johnston's organ
JO-A neurons: Subgroup-A JO neurons
JO-B neurons: Subgroup-B JO neurons
JO-D neurons: Subgroup-D JO neurons
AMMC: Antennal mechanosensory and motor center
PBS: Phosphate buffered saline
5-HT: 5-hydroxytryptamine
ChAT: Choline acetyltransferase
GABA: Gamma amino butyric acid
TNT: Tetanus toxin
3D: Three dimensional
CMTK: Computational Morphometry Toolkit
A-D: anterior-dorsal
P-D: posterior-dorsal
A-V: anterior-ventral
P-V: posterior-ventral
O-E: outer-edge
BK: Ca^2+^-activated K^+^
*slo*: *slowpoke*
Df: degree of freedom
Sq: square.
